# Predicting histologic differentiation of solitary hepatocellular carcinoma up to 5 cm on gadoxetate disodium-enhanced MRI

**DOI:** 10.1186/s13244-022-01354-w

**Published:** 2023-01-08

**Authors:** Ting Yang, Hong Wei, Yuanan Wu, Yun Qin, Jie Chen, Hanyu Jiang, Bin Song

**Affiliations:** 1 Department of Radiology, West China Hospital, Sichuan University, No. 37 Guoxue Alley, Chengdu, 610041 China; 2 Big Data Research Center, University of Electronic Science and Technology of China, Chengdu, 610000 Sichuan China; 3 Department of Radiology, Sanya People’s Hospital, Sanya, Hainan China

**Keywords:** Carcinoma (Hepatocellular), Histologic differentiation, Gadolinium ethoxybenzyl DTPA, Magnetic resonance imaging

## Abstract

**Background:**

To establish a preoperative score based on gadoxetate disodium-enhanced magnetic resonance imaging (EOB-MRI) and clinical indicators for predicting histologic differentiation of solitary HCC up to 5 cm.

**Methods:**

From July 2015 to January 2022, consecutive patients with surgically proven solitary HCC measuring ≤ 5 cm at preoperative EOB-MRI were retrospectively enrolled. All MR images were independently evaluated by two radiologists who were blinded to all clinical and pathologic information. Univariate and multivariate logistic regression analyses were performed to identify significant clinicoradiological features associated with poorly differentiated (PD) HCC, which were then incorporated into the predictive score. The predictive score was validated in an independent validation set by area under the receiver operating characteristic curve (AUC), sensitivity, specificity, and accuracy.

**Results:**

A total of 182 patients were included, 42 (23%) with PD HCC. According to the multivariate analysis, marked hepatobiliary phase hypointensity (odds ratio [OR], 9.98), LR-M category (OR, 5.60), and serum alpha-fetoprotein (AFP) level > 400 ng/mL (OR, 3.58) were incorporated into the predictive model; the predictive score achieved an AUC of 0.802 and 0.830 on the training and validation sets, respectively. The sensitivity, specificity, and accuracy of the predictive score were 66.7%, 85.7%, and 81.3%, respectively, on the training set and 66.7%, 81.0%, and 77.8%, respectively, on the validation set.

**Conclusion:**

The proposed score integrating two EOB-MRI features and AFP level can accurately predict PD HCC in the preoperative setting.

**Supplementary Information:**

The online version contains supplementary material available at 10.1186/s13244-022-01354-w.

## Key points

- EOB-MRI features help capture the characteristics of tumor biology and heterogeneity.
- EOB-MRI-based HCC differentiation score allowed accurate assessment of poor tumor differentiation preoperatively.
- This scoring system might be useful for prompting tailored treatment selection.

**Supplementary Information:**

The online version contains supplementary material available at 10.1186/s13244-022-01354-w.

## Background

Primary liver cancer is the sixth most common malignancy and the fourth leading cause of cancer-related death globally, and hepatocellular carcinoma (HCC) accounts for around 75–85% of these cases [1]. Liver resection constitutes the backbone for curative treatment of HCC in patients with preserved liver function. However, its application is dampened by high incidence of postoperative recurrence that approaches 60–70% at 5 years [2].

Histologic differentiation is an established prognostic indicator in HCC. In specific, poorly differentiated HCC (PD HCC) has been associated with worse overall survival and/or disease-free survival after liver resection [3], liver transplantation [4], and radiofrequency ablation [5]. Therefore, accurate assessment of tumor differentiation, particularly in the preoperative stage, can help tailor individualized treatment decision-making and improve postoperative survival. Nevertheless, the degree of differentiation is accessible only through histologic examination following invasive biopsy or surgery, which is prone to sampling errors or can only be evaluated postoperatively.

Fortunately, imaging techniques can provide important information regarding tumor biology and heterogeneity in a noninvasive manner. For degree of differentiation, studies have shown that tumor signal intensity in the hepatobiliary phase (HBP) of gadoxetate disodium-enhanced magnetic resonance imaging (EOB-MRI) was associated with histologic grade of HCC [6–9]. Other imaging features, including the “washout” appearance [10], lower tumor-to-liver enhancement ratio in early arterial phase, absence of “capsule,” arterial phase peritumoral hyperenhancement [11], and peritumoral hypointensity in HBP, were also reported to correlate with moderately differentiated (MD) or PD HCC [9].

Although results so far have been promising, the evidence qualities of previous studies were limited by potentially biased radiology-pathology correlation, as most of these studies did not pose strict restrictions on tumor number and size [7, 9, 11]. Nevertheless, considering the markedly heterogenous nature of HCC (especially in multifocal and/or large tumors) [12, 13], sampling errors may have greatly impacted the results. For example, multifocal HCCs may have distinct degrees of differentiation among tumors, but most prior work only evaluated the dominant tumors. As for first-line curative-intent treatment options, solitary HCC ≤ 5 cm was within the Milan criteria, by considering possible influence of tumor differentiation on prognosis, it may help prompting tailored treatment selection among transplant, locoregional treatment, and resection. Therefore, the imaging indicators of tumor differentiation in solitary HCC ≤ 5 cm remain to be defined.

Thus, this study aimed to establish an easy-to-use score based on preoperative EOB-MRI and clinical indicators for predicting histologic differentiation of solitary HCC up to 5 cm.

## Materials and methods

This single-center retrospective study was approved by the institutional review boards at West China Hospital, Sichuan University, and the requirements to obtain written informed consent were waived.

### Patients

From July 2015 to January 2022, consecutive patients were enrolled according to the following inclusion criteria: (1) age ≥ 18 years; (2) with history of chronic hepatitis B viral infection and/or liver cirrhosis not attributable to congenital or vascular abnormalities; (3) undergoing EOB-MRI within 1 month prior to liver resection; (4) with surgically proven single HCC measuring ≤ 5 cm at preoperative EOB-MRI; and (5) with complete baseline clinical information (detailed below) within 2 weeks before surgery.

The exclusion criteria were as follows: (1) any previous treatment for HCC; (2) the postoperative pathology report was inadequate for determining tumor differentiation; and (3) the MR images were of insufficient quality (e.g., severe motion artifact) for image analysis.

Baseline preoperative clinical information, including patient demographics, underlying liver diseases, presence or absence of cirrhosis, Child–Pugh classes, and key laboratory data (e.g., alpha-fetoprotein [AFP], carbohydrate antigen 199 [CA199], alanine aminotransferase [ALT], aspartate aminotransferase [AST], total bilirubin [TBIL], albumin [ALB]), was recorded.

### Image acquisition

The EOB-MR images were acquired on various 1.5 T or 3.0 T MR systems. The MRI sequences included: T2-weighted imaging (T2WI); diffusion-weighted imaging (DWI) with apparent diffusion coefficient (ADC) maps; in- and opposed-phase T1-weighted imaging; and dynamic T1-weighted imaging before and after injection of gadoxetate disodium (Xianai®, Zhengdatianqing Pharmaceutical Group; Primovist®, Bayer Schering Pharma AG) in the late arterial phase (AP), portal venous phase (PVP), transitional phase (TP), and HBP. Detailed imaging acquisition parameters are shown in Additional file 1: A1 and Table S1.

### Image analysis

All de-identified MR images were independently reviewed by two fellowship-trained radiologists (with 5 and 7 years of experiences in liver MRI, respectively). The reviewers were aware that all patients had HCC but were blinded to all clinical, laboratory, and pathologic results. All disagreements between the two reviewers were resolved by a senior fellowship-trained radiologist who had over 20 years of experience in liver MRI.

The reviewers independently evaluated all the lesions regarding the presence/absence/degree of imaging features which had been reported to correlate with the degree of tumor differentiation, tumor burden, and HCC aggressiveness, including: (1) imaging features correlated with tumor differentiation (e.g., HBP signal intensity, degree of diffusion restriction); (2) all Liver Imaging Reporting and Data System (LI-RADS) version 2018 major and ancillary features (except for those related to growth and ultrasound visibility), LR-M features, TIV features, and LI-RADS category [14]; (3) imaging features profiling peritumoral changes (e.g., peritumoral HBP hypointensity [15], peritumoral T2WI hyperintensity, peritumoral biliary ductal dilation); and (4) other reported prognostic imaging features (e.g., intratumoral artery, non-smooth tumor margin, complete “capsule,” tumor growth pattern). The definitions and illustrations of the imaging features are detailed in Additional file 1: Table S2.

### Histopathology

Information on tumor differentiation was extracted from routine pathological reports as the reference standard for tumor differentiation, which was recorded by another radiologist without knowing patient’s imaging and clinical data. Based on the standard operation procedure of our institution, a 7-site sampling procedure was performed to ensure adequate and reliable assessment of tumor differentiation. In specific, one piece of tissue was each sampled at the transition area between tumor and surrounding liver tissues at a ratio of 1:1 at 12, 3, 6 and 9 o’clock from the less bleeding and necrotic areas. One piece was sampled within the tumor area free from bleeding and necrosis, and one piece each was sampled, respectively, from proximal (≤ 1 cm to the tumor) and distant liver parenchyma (> 1 cm to the tumor). Tumor differentiation was determined according to the World Health Organization (WHO) criteria [16]. The higher pathological grade was recorded when the evaluated HCC had mixed tumor grades.

### Statistical analysis

Continuous variables were presented as mean ± standard deviation or median (interquartile range [IQR]) and analyzed with either the Student’s t test or the Mann–Whitney U test, whereas categorical variables were presented as the numbers of cases (percentages) and analyzed with either the Chi-square test or the Fisher 's exact test, where applicable.

Interobserver agreement between the two radiologists was assessed by calculating the intraclass correlation coefficient (ICC) for continuous imaging features, the Cohen’s κ value for binary imaging features, and the weighted κ value for categorical/ordinal imaging features, respectively.

#### Development of the predictive score for tumor differentiation

All eligible patients were randomly assigned into a training set and an independent validation set at a ratio of 7:3, while guaranteeing that the distributions of poorly and non-poorly differentiated HCCs between two sets were comparable.

In the training dataset, significant clinical and imaging predictors of PD HCC were selected by univariate logistic regression analysis. Afterward, controlling for patient age, gender, and underlying liver cirrhosis, all predictors with *p* < 0.1 at univariate logistic regression analysis were fit into a multivariate logistic regression model with backward stepwise method based on fivefold cross-validation, and the best-fit feature combination was obtained using the Akaike Information Criterion. To improve clinical utility and model simplicity, continuous variables were converted to categorical or binary ones according to normal range or clinical relevance. Inter-variable correlations were estimated by the pairwise Spearman’s correlation analysis; when significant collinearity was observed, variables with the largest odds ratio (OR) at univariate logistic regression analysis were kept for further analysis. An *HCC differentiation score* was developed based on the significant predictors at multivariate logistic regression analysis weighted by their *β* regression coefficients, with the largest *β* coefficient scaled as 10 points. The Youden’s index was used to analyze the receiver operating characteristic curve to determine the optimal threshold of the *HCC differentiation score* for predicting PD HCC.

#### Validation of the predictive score for tumor differentiation

The discriminative accuracy of the *HCC differentiation score* was evaluated with area under the receiver operating characteristic curve (AUC), sensitivity, specificity, positive predictive value (PPV), negative predictive value (NPV), and accuracy. Model calibration was assessed by the calibration curve with the Hosmer–Lemeshow test. Decision curve analysis was performed to investigate the clinical usefulness of the *HCC differentiation score* by quantifying the net benefits at different threshold probabilities.

The R software (version 3.5.1) and SPSS (version 26) were used to perform the statistical analysis. Two- tailed *p* ≤ 0.05 was considered statistically significant.

## Results

### Patient characteristics

**Fig. 1:**
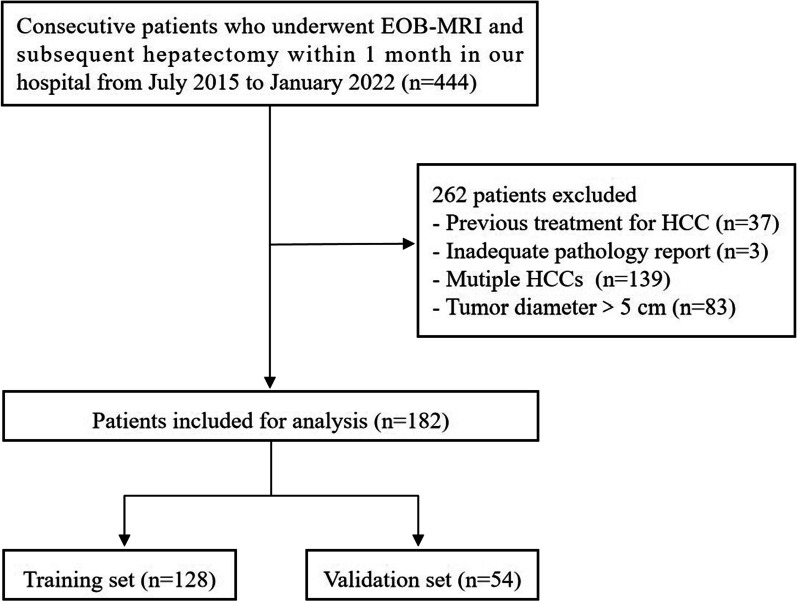
Flowchart of patient selection. EOB-MRI, gadoxetate disodium-enhanced magnetic resonance imaging; HCC, hepatocellular carcinoma

A total of 182 patients (mean age ± standard deviation, 52.9 ± 11.2; range, 28–75; 145 men) were included in this study, among whom 128 and 54 patients were divided into the training and validation sets, respectively (Fig. 1).

Baseline clinical characteristics are presented in Table 1; frequencies of EOB-MRI features are summarized in Table 2; comparisons in baseline clinical characteristics of patients between the training and validation sets are shown in the Additional file 1: Table S3, and interobserver agreement for all imaging features and LI-RADS categories are detailed in Additional file 1: Table S4.

**Table 1:** Baseline clinical characteristics of patients

	Training set (*n* = 128)			Validation set (*n* = 54)		
	WD/MD HCC (*n* = 98)	PD HCC (*n* = 30)	*p* value	WD/MD HCC (*n* = 42)	PD HCC (*n* = 12)	*p* value
*Patient demographics*						
Age, years	52.1 ± 11.6	54.9 ± 11.1	0.645	52.8 ± 9.94	54.5 ± 13.0	0.184
Sex			0.941			1.000
Male	79 (80.6)	24 (80.0)		33 (78.6)	9 (75.0)	
Female	19 (19.4)	6 (20.0)		9 (21.4)	3 (25.0)	
*Underlying liver diseases*						
Chronic hepatitis B	90 (91.8)	29 (96.7)	0.619	41 (97.6)	11 (91.7)	0.398
Chronic hepatitis C	0 (0.0)	0 (0.0)	–	0 (0.0)	1 (8.3)	–
Chronic hepatitis B and C	2 (2.0)	0 (0.0)	–	0 (0.0)	0 (0.0)	–
Alcohol	2 (2.0)	0 (0.0)	–	0 (0.0)	0 (0.0)	–
NAFLD	1 (1.0)	0 (0.0)	–	1 (2.4)	0 (0.0)	–
Others	3 (3.1)	1 (3.3)	–	0 (0.0)	0 (0.0)	–
Cirrohsis	60 (61.2)	19 (63.3)	0.835	24 (57.1)	6 (50.0)	0.661
*Child–Pugh stage*			0.554			–
A	96 (98)	29 (96.7)		42 (100.0)	12 (100.0)	
B	2 (2)	1 (3.3)		0 (0.0)	0 (0.0)	
*Tumor marker*						
AFP, ng/mL			0.010*			0.058
≤ 400	81 (82.7)	18 (60.0)		39 (92.9)	8 (66.7)	
> 400	17 (17.3)	12 (40.0)		3 (7.1)	4 (33.3)	
CA199, U/mL			0.332			0.661
≤ 30	77 (78.6)	21 (70.0)		36 (85.7)	9 (75.0)	
> 30	21 (21.4)	9 (30.0)		6 (14.3)	3 (25.0)	
CEA, ng/mL			1.000			–
≤ 5	90 (91.8)	28 (93.3)		42 (100.0)	12 (100.0)	
> 5	8 (8.2)	2 (6.7)		0 (0.0)	0 (0.0)	
*Laboratory index*						
TBIL, µmol/L			1.000			–
≤ 40	96 (98.0)	30 (100.0)		42 (100.0)	12 (100.0)	
> 40	2 (2.0)	0 (0.0)		0 (0.0)	0 (0.0)	
ALT, U/L			0.515			0.610
≤ 35	62 (63.3)	17 (56.7)		21 (50.0)	7 (58.3)	
> 35	36 (36.7)	13 (43.3)		21 (50.0)	5 (41.7)	
AST, U/L			0.103			0.543
≤ 35	71 (72.4)	17 (56.7)		29 (69.0)	10 (83.3)	
> 35	27 (27.6)	13 (43.3)		13 (31.0)	2 ((16.7)	
ALB, g/L			0.867			0.640
≥ 40	77 (78.6)	24 (80.0)		33 (78.6)	8 (66.7)	
< 40	21 (21.4)	6 (20.0)		9 (21.4)	4 (33.3)	
PLT, 10^9^/L			0.845			1.000
≥ 125	47 (48.0)	15 (50.0)		17 (40.5)	5 (41.7)	
< 125	51 (52.0)	15 (50.0)		25 (59.5)	7 (58.3)	
PT, s			0.636			0.012*
≤ 12.8	87 (88.8)	25 (83.3)		39 (92.9)	7 (58.3)	
> 12.8	11 (11.2)	5 (16.7)		3 (7.1)	5 (41.7)	

Data are expressed as *n* (%) or median (interquartile range). Continuous variables are presented as mean ± standard deviation. These variables were compared using t-test or nonparametric Mann–Whitney tests. Categorical variables are the number of patients, with percentages in parentheses. These variables were compared using the chi-square or Fisher exact test

WD, well differentiated; MD, moderately differentiated; PD, poorly differentiated; HBV; hepatitis B virus; AFP, a-fetoprotein; CA199, Carbohydrate antigen 199; CEA, carcinoembryonic antigen; TBIL, total bilirubin; ALT, alanine aminotransferase; AST, aspartate aminotransferase; ALB, albumin; PLT, platelet count; PT, prothrombin time

*Variables are statistically significant

**Table 2:** Frequencies of EOB-MRI features

Variable	Training set (*n* = 128)			Validation set (*n* = 54)		
	WD/MD HCC (*n* = 98)	PD HCC (*n* = 30)	*p* value	WD/MD HCC (*n* = 42)	PD HCC (*n* = 12)	*p* value
LI-RADS v2018 feature^a^						
Size, cm	2.6 (1.6–3.6)	2.8 (1.7–4.0)	0.200	2.8 (1.8–3.8)	2.4 (1.6–3.2)	0.317
Nonrim arterial phase hyperenhancement	90 (91.8)	25 (83.3)	0.316	38 (90.5)	11 (91.7)	1.000
Nonperipheral "washout"	87 (88.8)	26 (86.7)	1.000	8 (19.0)	0 (0.0)	0.239
Enhancing "capsule"	71 (72.4)	21 (70.0)	0.794	32 (76.2)	9 (75.0)	1.000
Corona enhancement	24 (24.5)	7 (23.3)	0.897	12 (28.6)	3 (25.0)	1.000
Fat sparing in solid mass	8 (8.2)	5 (16.7)	0.316	0 (0.0)	1 (8.3)	0.222
Diffusion restriction	97 (99.0)	30 (100.0)	1.000	42 (100.0)	12 (100.0)	–
Mild-moderate T2 hyperintensity	95 (96.9)	30 (100.0)	1.000	41 (97.6)	11 (91.7)	0.398
Iron sparing in solid mass	24 (24.5)	4 (13.3)	0.196	9 (21.4)	2 (16.7)	1.000
Transitional phase hypointensity	93 (94.9)	30 (100.0)	0.469	40 (95.2)	12 (100.0)	1.000
Hepatobiliary phase hypointensity	92 (93.9)	29 (96.7)	0.897	42 (100.0)	12 (100.0)	–
Nonenhancing "capsule"	13 (13.3)	2 (6.7)	0.510	7 (16.7)	1 (8.3)	0.798
Nodule-in-nodule	24 (24.5)	9 (30.0)	0.546	6 (14.3)	2 (16.7)	1.000
Mosaic architecture	16 (16.3)	6 (20.0)	0.641	4 (9.5)	2 (16.7)	0.862
Fat in mass, more than adjacent liver	41 (41.8)	14 (46.7)	0.640	23 (54.8)	7 (58.3)	0.826
Blood products in mass	19 (19.4)	4 (13.3)	0.450	5 (11.9)	3 (25.0)	0.506
Iron in mass, more than liver	3 (3.1)	1 (3.3)	1.000	0 (0.0)	0 (0.0)	–
Marked T2 hyperintensity	2 (2.0)	0 (0.0)	1.000	0 (0.0)	1 (8.3)	0.222
Hepatobiliary phase isointensity	5 (5.1)	1 (3.3)	1.000	0 (0.0)	0 (0.0)	–
Tumor in vein	2 (2.0)	1 (3.3)	0.554	1 (2.4)	0 (0.0)	1.000
Rim arterial phase hyperenhancement	3 (3.1)	5 (16.7)	0.024*	4 (9.5)	0 (0.0)	0.564
Peripheral "washout"	1 (1.0)	0 (0.0)	1.000	0 (0.0)	0 (0.0)	–
Delayed central enhancement	0 (0.0)	0 (0.0)	–	1 (2.4)	0 (0.0)	1.000
Targetoid TP or HBP appearance	1 (1.0)	1 (3.3)	0.415	0 (0.0)	0 (0.0)	–
Infiltrative appearance	12 (12.2)	3 (10.0)	0.992	2 (4.8)	0 (0.0)	1.000
Marked diffusion restriction	32 (32.7)	16 (53.3)	0.041*	13 (31.0)	6 (50.0)	0.381
Necrosis or severe ischemia	15 (15.3)	2 (6.7)	0.361	6 (14.3)	2 (16.7)	1.000
LR-M category	3 (3.1)	6 (20.0)	0.006*	3 (7.1)	0 (0.0)	1.000
LR-TIV category	2 (2.0)	1 (3.3)	0.554	1 (2.4)	0 (0.0)	1.000
LR-3 category	1 (1.0)	0 (0.0)	1.000	0 (0.0)	0 (0.0)	–
LR-4 category	9 (9.2)	2 (6.7)	0.954	3 (7.1)	1 (8.3)	1.000
LR-5 category	83 (84.7)	21 (70.0)	0.071	35 (83.3)	11 (91.7)	0.798
Other imaging feature						
Liver surface retraction	2 (2.0)	0 (0.0)	1.000	0 (0.0)	1 (8.3)	0.222
Adjacent biliary dilatation	1 (1.0)	1 (3.3)	0.415	0 (0.0)	1 (8.3)	0.222
Radiologic cirrhosis	60 (61.2)	19 (63.3)	0.835	24 (57.1)	6 (50.0)	0.661
Bilobar involvement	4 (4.1)	3 (10.0)	0.430	4 (9.5)	0 (0.0)	0.564
Internal artery	13 (13.3)	3 (10.0)	0.875	5 (11.9)	1 (8.3)	1.000
Non-smooth tumor margin	49 (50.0)	17 (56.7)	0.523	21 (50.0)	4 (33.3)	0.307
Peritumoral hypointensity on PVP	18 (18.4)	4 (13.3)	0.523	3 (7.1)	2 (16.7)	0.661
Peritumoral hypointensity on TP	16 (16.3)	3 (10.0)	0.576	3 (7.1)	2 (16.7)	0.661
Peritumoral hypointensity on HBP	25 (25.5)	11 (36.7)	0.234	7 (16.7)	1 (8.3)	0.798
Marked HBP hypointensity	12 (12.2)	19 (63.3)	< 0.001*	4 (9.5)	8 (66.7)	< 0.001*
Single nodular type growth	71 (72.4)	19 (63.3)	0.339	15 (35.7)	4 (33.3)	1.000
Peritumoral hyperintensity on T2WI	7 (7.1)	5 (16.7)	0.227	2 (4.8)	0 (0.0)	1.000
Non-hypervascular HBP hypointense						
Nodules	19 (19.4)	8 (26.7)	0.393	17 (40.5)	4 (33.3)	0.911

Data are expressed as the frequencies of MRI features, with percentages in parentheses

EOB-MRI, gadoxetic acid–enhanced magnetic resonance imaging; LI-RADS/LR, Liver Imaging Reporting and Data System; HCC, hepatocellular carcinoma; WD, well differentiated; MD, moderately differentiated; PD, poorly differentiated; HBV; T2WI, T2-weighted imaging; PVP, portal venous phase; TP, transitional phase; HBP, hepatobiliary phase

*Variables are statistically significant

^a^LI-RADS v2018 features correlated with growth or ultrasound visibility were not assessed due to lack of prior and concurrent ultrasound examinations

On the training set, PD HCC was pathologically confirmed in 30 patients (23.4%; 30/128). No significant differences in baseline clinical parameters were observed between patients with PD HCC and those with well differentiated (WD) or MD HCC (*p* = 0.103–1.000), except for serum AFP level (*p* = 0.010). Moreover, rim arterial phase hyperenhancement, marked diffusion restriction, LR-M category and marked HBP hypointensity were significantly more frequent in PD HCC than in WD/MD HCC. Other EOB-MRI features were similar in both groups (*p* = 0.071–1.000).

On the validation set, PD HCC was pathologically confirmed in 12 patients (22.2%; 12/54). There were no significant differences in baseline clinical variables between patients with PD HCC and those with WD/MD HCC (*p* = 0.058–1.000), except for PT (*p* = 0.012). Additionally, the frequency of marked HBP hypointensity was significantly higher in PD HCC compared with WD/MD HCC. Other EOB-MRI features were not significantly different in both groups (*p* = 0.222–1.000).

Elevated serum carcinoembryonic antigen (> 5 ng/mL) was more frequently observed for patients in the training dataset (7.8% vs. 0%, *p* = 0.035) than those in the validation dataset. No difference in other baseline clinical characteristics was detected between the training and testing dataset (*p* = 0.134–0.784).

#### Development of the HCC differentiation score on the training set

**Fig. 2:**
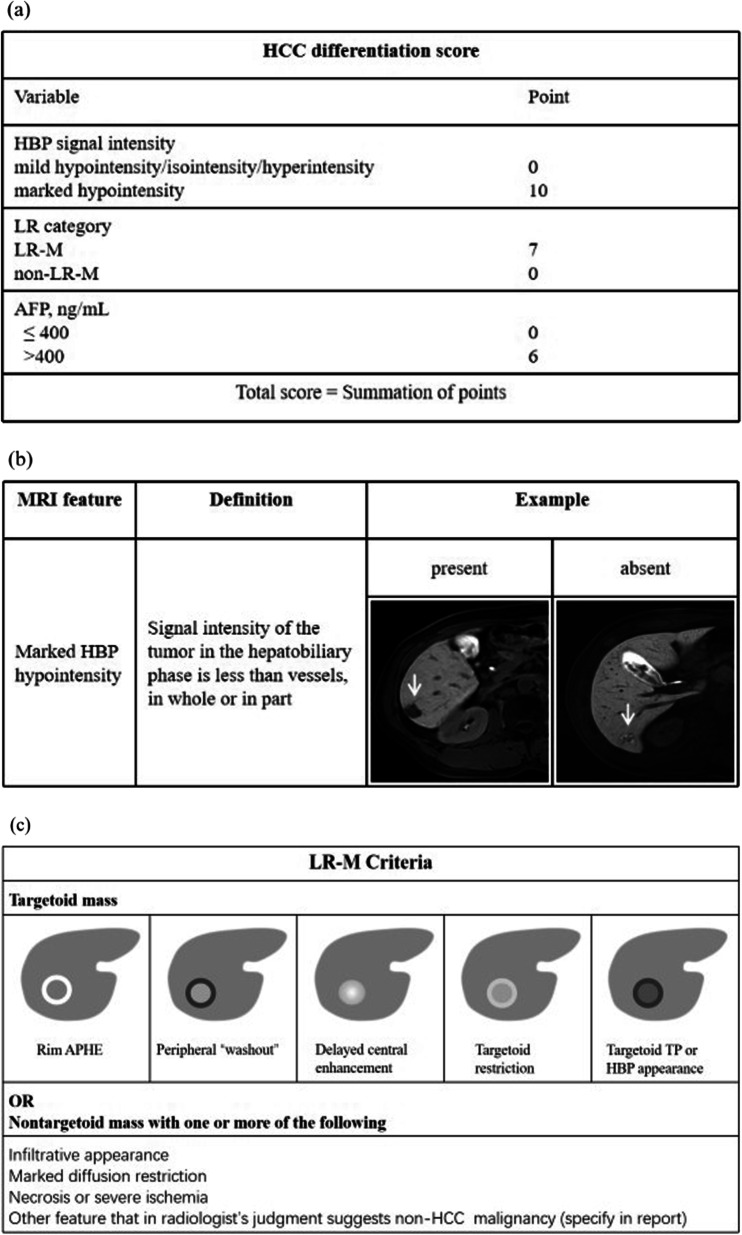
**a** Illustration of the HCC differentiation score; **b** definitions and representative images of the MRI feature associated with the HCC differentiation score; **c** illustration of LR-M criteria. HCC, hepatocellular carcinoma; AFP, serum alpha-fetoprotein; HBP, hepatobiliary phase; LR, Liver Imaging Reporting and Data System

**Fig. 3:**
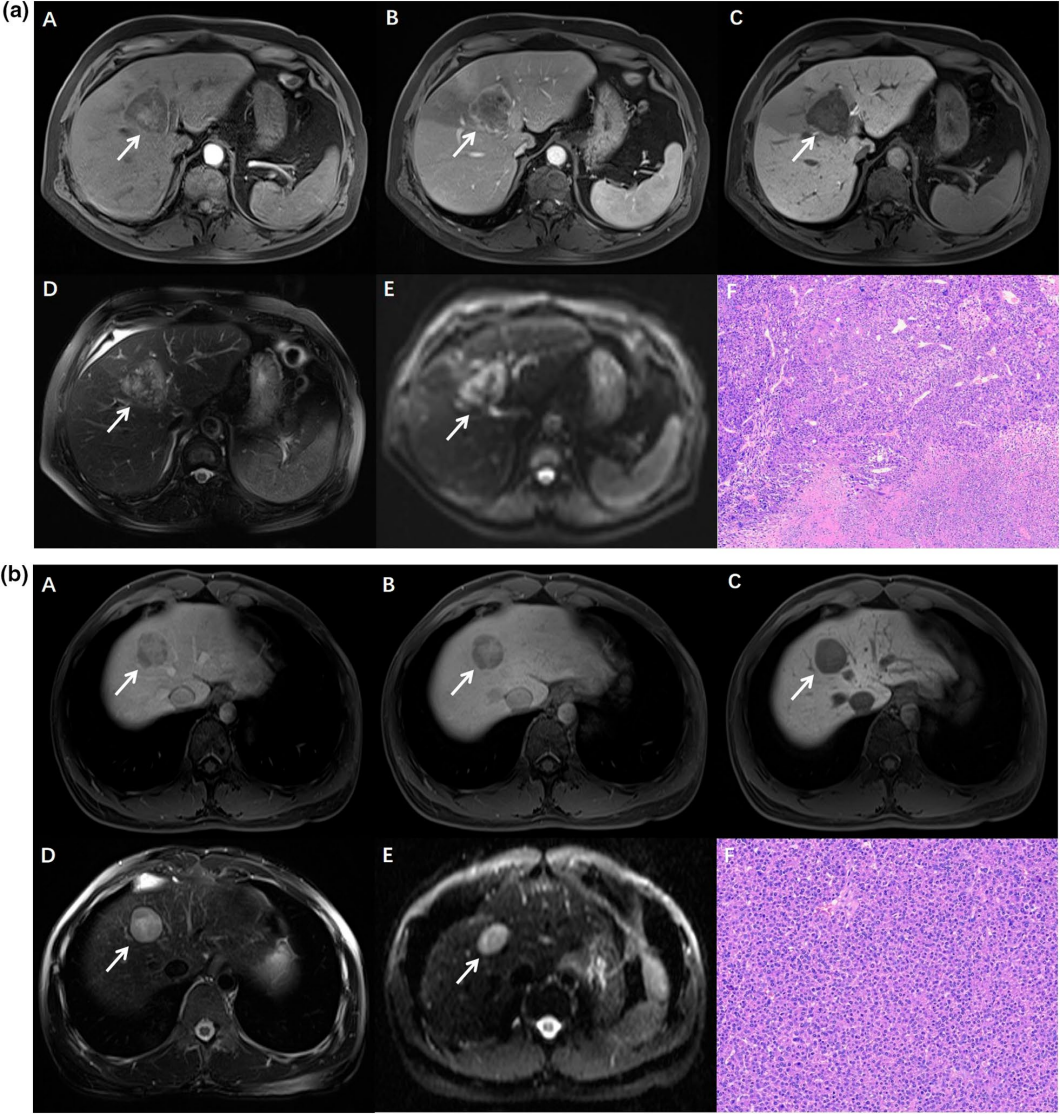
Typical cases of poorly and moderately differentiated hepatocellular carcinoma (HCC). (**a**) A 61-year-old man with poorly differentiated HCC. (A) Rim hyperenhancement is visible on the arterial phase (arrow). (B) The portal venous phase shows an incomplete enhancing tumor capsule (arrow). (C) The signal intensity of the lesion on the hepatobiliary phase is less than vessels, showing marked hypointensity, and peritumoral hypointensity is also seen on the hepatobiliary phase (arrow). (D) Axial T2-weighted imaging shows a hyperintense signal lesion (arrow). (E) Diffusion weighted imaging shows a marked-restricted-diffusion lesion (arrow). (F) Photomicrograph shows the poorly differentiated HCC (hematoxylin–eosin stain; original magnification, × 100). (**b**) A 31-year-old man with moderately differentiated HCC. (A) Non-rim hyperenhancement is visible on the arterial phase (arrow). (B) The tumor present washout on the portal venous phase (arrow). (C)The lesion shows mild hypointensity on the hepatobiliary phase (arrow). (D) Axial T2-weighted imaging shows a hyperintense signal lesion (arrow). (E) Diffusion-weighted imaging shows a marked-restricted-diffusion lesion (arrow). (F) Photomicrograph shows the moderately differentiated HCC (hematoxylin–eosin stain; original magnification, 100)

Significant clinical and imaging predictors of PD HCC, including patient demographics, etiology, Child–Pugh stage, tumor markers (i.e., AFP, CA199, CEA), laboratory indexes (e.g., ALT, AST, TBIL), LI-RADS v2018 feature and other imaging features which had been reported to correlate with the degree of tumor differentiation, tumor burden, and HCC aggressiveness (e.g., marked HBP hypointensity, peritumoral hypointensity in HBP, and complete capsule), were analyzed in the univariate analysis. And it identified 6 variables predictive of PD HCC on the training set, including LR-M category (OR, 7.92; *p* = 0.005), marked HBP hypointensity (OR, 12.38; *P* < 0.001), serum AFP level > 400 ng/mL (OR, 3.18; *p* = 0.012), rim arterial phase hyperenhancement (OR, 6.33; *p* = 0.016), and marked diffusion restriction (OR, 3.26; *p* = 0.043). According to the multivariate analysis (Table 3), LR-M category (odds ratio [OR], 5.60; *p* = 0.054), marked hepatobiliary phase hypointensity (OR, 9.98; *P* < 0.001), and serum AFP level > 400 ng/mL (OR, 3.58; *p* = 0.021) were incorporated into the HCC differentiation score and are illustrated in Fig. 2. The total score was calculated by adding the individual points of each variable, ranging from 0 to 23 points. According to Youden’s index, the optimal threshold for predicting PD HCC was 6.5 points. Typical patients with PD HCC or MD HCC are shown in Fig. 3.

**Table 3:** Univariable and multivariable logistic regression analyses of predictors for histological differentiation of hepatocellular carcinoma on training set

Characteristics	Univariable analysis		Multivariable analysis		
	Odds ratio (95%CI)	*p* value	Odds ratio (95%CI)	*β* Coefficient	*p* value
Patient demographics					
Age, year	1.02 (0.99, 1.06)	0.234	–	–	–
Sex (male)	0.96 (0.35, 2.68)	0.941	–	–	–
Etiology (HBV-related)	0.39 (0.05, 3.23)	0.381	–	–	–
Child–Pugh stage (A vs. B)	1.66 (0.14, 18.92)	0.685	–	–	–
Tumor markers					
AFP > 400 ng/mL	3.18 (1.29, 7.80)	0.012	3.58 (1.22, 10.54)	1.28	0.021
CA199 > 30 U/mL	1.57 (0.63, 3.94)	0.334	–	–	–
CEA > 5 ng/mL	0.80 (0.16, 4.01)	0.790	–	–	–
Laboratory indexes					
TBIL > 28 µmol/L	1.33 (0.24, 7.22)	0.742	–	–	–
ALT > 35 U/L	1.32 (0.57, 3.02)	0.516	–	–	–
AST > 35 U/L	2.01 (0.86, 4.69)	0.106	–	–	–
ALB > 55 g/L	1.09 (0.39, 3.01)	0.867	–	–	–
PLT > 125 × 10^9^/L	1.09 (0.48, 2.46)	0.845	–	–	–
PT > 12.8 s	1.58 (0.50, 4.98)	0.433	–	–	–
LI-RADS v2018 feature					
Size, cm	1.21 (0.82, 1.77)	0.337	–	–	–
Nonrim arterial phase hyperenhancement	2.25 (0.68, 7.49)	0.186	–	–	–
Nonperipheral “washout”	0.82 (0.24, 2.80)	0.754	–	–	–
Enhancing "capsule"	0.89 (0.36, 2.18)	0.794	–	–	–
Corona enhancement	0.94 (0.36, 2.46)	0.897	–	–	–
Fat sparing in solid mass	2.25 (0.68, 7.49)	0.186	–	–	–
Diffusion restriction	1,780,766.87 (0, + ∞)	0.992	–	–	–
Mild–moderate T2 hyperintensity	4,942,535.01 (0, + ∞)	0.991	–	–	–
Iron sparing in solid mass	0.47 (0.15, 1.50)	0.203	–	–	–
Transitional phase hypointensity	5,048,826.06 (0, + ∞)	0.989	–	–	–
Hepatobiliary phase hypointensity	1.89 (0.22, 16.36)	0.563	–	–	–
Nonenhancing “capsule”	0.47 (0.10, 2.20)	0.335	–	–	–
Nodule in nodule	1.32 (0.53, 3.27)	0.547	–	–	–
Mosaic architecture	1.28 (0.45, 3.63)	0.641	–	–	–
Fat in mass, more than adjacent liver	1.22 (0.53, 2.77)	0.640	–	–	–
Blood products in mass	0.64 (0.20, 2.05)	0.453	–	–	–
Iron in mass, more than liver	1.09 (0.11, 10.90)	0.940	–	–	–
Marked T2 hyperintensity	55,576,658.67 (0, + ∞)	0.989	–	–	–
Hepatobiliary phase isointensity	0.64 (0.07,5.71)	0.691	–	–	–
Tumor in vein	1.66 (0.14, 18.92)	0.685	–	–	–
Rim arterial phase hyperenhancement	6.33 (1.42, 28.32)	0.016	–	–	–
Peripheral “washout”	1.22 (0.36, 4.14)	0.754	–	–	–
Targetoid TP or HBP appearance	3.34 (0.20, 55.15)	0.398	–	–	–
Infiltrative appearance	0.80 (0.21, 3.03)	0.738	–	–	–
Marked diffusion restriction	2.36 (1.03, 5.42)	0.043	–	–	–
Necrosis or severe ischemia	0.40 (0.09, 1.84)	0.236	–	–	–
LR-M category	7.92 (1.85, 33.97)	0.005	5.60 (0.97–32.36)	1.72	0.054
Other imaging feature					
Liver surface retraction	55,576,658.67 (0, + ∞)	0.989	–	–	–
Adjacent biliary dilatation	3.34 (0.20, 55.15)	0.398	–	–	–
Radiologic cirrhosis	1.09 (0.47, 2.55)	0.835	–	–	–
Bilobar involvement	2.61 (0.55, 12.39)	0.227	–	–	–
Internal artery	0.73 (0.19, 2.74)	0.637	–	–	–
Non-smooth tumor margin	1.31 (0.57, 2.98)	0.523	–	–	–
Peritumoral hypointensity in PVP	0.68 (0.21, 2.20)	0.524	–	–	–
Peritumoral hypointensity in TP	0.57 (0.15, 2.11)	0.399	–	–	–
Peritumoral hypointensity in HBP	1.69 (0.71, 4.04)	0.237	–	–	–
Marked HBP hypointensity	12.38 (4.75, 32.24)	< 0.001	9.98 (3.63, 27.46)	2.30	< 0.001
Single nodular type growth	1.52 (0.64, 3.61)	0.341	–	–	–
Peritumoral hyperintensity on T2WI	2.60 (0.76, 8.90)	0.128	–	–	–
Non-hypervascular HBP hypointense nodules	1.51 (0.58, 3.92)	0.395	–	–	–

Data are presented as median (95% confidence intervals)

HCC, hepatocellular carcinoma; WD, well differentiated; MD, moderately differentiated; PD, poorly differentiated; HBV; hepatitis B virus; AFP, a-fetoprotein; CA199, Carbohydrate antigen 199; CEA, carcinoembryonic antigen; TBIL, total bilirubin; ALT,alanine aminotransferase; AST, aspartate aminotransferase; ALB, albumin; PLT, platelet count; PT, prothrombin time. EOB-MRI, gadoxetic acid–enhanced magnetic resonance imaging; LI-RADS/LR, Liver Imaging Reporting and Data System; T2WI, T2-weighted imaging; PVP, portal venous phase; TP, transitional phase; HBP, hepatobiliary phase

#### Score assessment and validation

On the training set, the HCC differentiation score exhibited an AUC of 0.802 (0.703–0.900) with sensitivity, specificity, PPV, NPV, and accuracy of 66.7%, 85.7%, 58.8%, 89.4%, 81.3%, respectively. Calibration plot demonstrated a good consistency between the score-predicted probabilities and the actual PD HCC estimates on the training set (*p* = 0.883; Additional file 1: Fig. S1a). Decision curves revealed that the predictive score provided a larger net benefit than that of assuming all patients had PD HCC when the threshold probability was greater than 0.1 on the training set (Additional file 1: Fig. S2a).

On the validation set, the predictive score yielded an AUC of 0.830 (0.693–0.966) with sensitivity, specificity, PPV, NPV and accuracy of 66.7%, 81.0%, 50.0%, 89.5%, 77.8%, respectively (Table 4). Calibration plot showed that the score-predicted probabilities were closely consistent with the actual PD HCC estimates on the validation set (*p* = 0.963; Additional file 1: Fig. S1b). In terms of the clinical utility, the predictive score exhibited a larger net benefit than that of assuming all patients had PD HCC when the threshold probability was greater than 0.08 on the validation set (Additional file 1: Fig. S2b).

**Table 4:** Diagnostic performance of models for predicting histological differentiation of hepatocellular carcinoma

	Training set	Validation set
AUC (95%CI)	0.802 (0.703–0.900)	0.830 (0.693–0.966)
Sensitivity	66.7% (20/30)	66.7% (8/12)
Specificity	85.7% (84/98)	81.0% (34/42)
PPV	58.8% (20/34)	50.00% (8/16)
NPV	89.4% (84/94)	89.5% (34/38)
ACC	81.3% (104/128)	77.8% (42/54)

Numbers in parentheses are 95% CIs, and numbers in brackets are raw data

AUC, receiver operating characteristic curve; PPV, positive predictive value; NPV, negative predictive value; ACC, accuracy

## Discussion

In patients with surgically-confirmed solitary HCC ≤ 5 cm, we found that two EOB-MR imaging features (marked hepatobiliary phase hypointensity and the LR-M category) along with serum AFP > 400 ng/mL were significantly associated with poor tumor differentiation. Based on these indicators, we developed and validated an easy-to-use scoring system which allowed accurate preoperative assessment of tumor differentiation.

Marked HBP hypointensity, defined as tumor signal intensity in the HBP lower than that of liver and similar to or lower than that of intrahepatic vessels, was present in 43 (24%) patients and significantly associated with poor tumor differentiation. This finding was in line with previous studies, in which lower tumor signal intensity in the HBP was associated with worse differentiation [7, 8]. One possible explanation for this outcome is that the expressions of organic anion transporters on the cell membrane, which are responsible for the uptake of gadoxetic disodium and signal intensity in the HBP, decrease gradually as the tumor de-differentiates [17]. However, the assessments of HBP signal intensity were mostly performed via quantitative analyses based on manual regions of interest placement in prior works [7, 8]. Despite allowing accurate quantifications of signal intensities, this approach may suffer from suboptimal generalizability, as signal intensity in the HBP is largely affected by vendor and acquisition parameter variations. In contrast, using intrahepatic vessels as a reference, we explored a reproducible and effective semi-quantitative way of measuring relative tumor signal intensity on HBP images, which could aid in preoperative evaluation of HCC differentiation.

The LR-M category is defined as lesions that are probably or definitely malignant but not HCC specific. Any targetoid appearances or nontargetoid mass with one or more features (including infiltrative appearance, marked diffusion restriction, necrosis or severe ischemia, and other feature that suggests non-HCC malignant), were considered to be sufficient for LR-M categorization [14]. And it was another imaging indicator of PD HCC. Our results were in consistent with the study of Shin et al., who found that the LR-M HCCs had poorer histologic differentiation than those LR-4/5 tumors [18]. In addition to worse differentiation, the LR-M category has also been reported to correlate with other aggressive pathomolecular characteristics of HCC, including increased stemness features (i.e., expression of CK19) [19], more pronounced macrotrabecular pattern, more frequent microvascular invasion and sinusoid-like microvascular pattern, and a more hypoxic and fibrotic tumor microenvironment [20]. As a result, LR-M category was widely-proved as an independent risk factor for worse prognosis in HCC [18, 21].

Serum AFP level > 400 ng/mL was the only laboratory indicator significantly associated with poor tumor differentiation in our study, which was in line with existing evidence. As the most established biomarker in HCC, higher serum AFP levels have been associated with increased tumor aggressiveness (e.g., worse tumor differentiation [22], increased incidence of microvascular invasion [23], macrotrabecular-massive subtype [20, 24], proliferative HCC [25]), and worse posttreatment prognosis [26–28]. Our work further confirmed its utility, alone and in combination with other imaging features, in identifying PD HCC.

Our findings had important clinical implications. Methodologically, considering marked intra- and intertumoral heterogeneity in HCC, satisfactory radiology–pathology correlation was achieved via the rigorous multi-site sampling method as we only enrolled patients with solitary tumors measuring ≤ 5 cm. Of note, identification of poorly-differentiated elements in small tumors might have been more challenging compared with tumors > 5 cm since these components are less frequently found in smaller HCCs [29]. However, our model still demonstrated AUCs over 0.80 on both the training and testing datasets. Moreover, all three indicators included in the final scoring system were identified as significantly associated with PD HCC in three out of the five folds of cross-validation during modeling. These outcomes further confirmed the robustness and effectiveness of our findings. In terms of clinical utility, all patients included in the current study were within the Milan criteria, of whom ablation, resection, and transplant are matched first-line curative-intent treatment options. In this context, the *HCC differentiation score* could serve as an effective therapeutic decision-making tool. Specifically, during the preoperative work-ups, our findings may help informing neoadjuvant treatment [30], as well as prompting resection over radiofrequency ablation even for small tumors less than 2 cm [31] in patients with PD HCCs according to the scoring system.

Several limitations of the current study should be noted. First, as a single-institutional retrospective study with relatively small sample size, no external validation was available to assess the performance and generalizability of our *HCC differentiation score*. Second, most of our enrolled patients had chronic hepatitis B virus infection. However, HCCs developed in non-HBV background (more prevalent in Western countries) harbor distinct pathomolecular characteristics from those in HBV patients; thus, our findings mandate further validations in HCC patients free of HBV infections. Third, aiming to propose an easy-to-use and interpretable scoring system for routine clinical adoptions, we only analyzed qualitative/semi-quantitative imaging indicators without consideration of complex and high-dimensional quantitative analyses. Another limitation is the relative subjectivity when evaluating imaging features, and the interreader agreement of some imaging features is poor in our work. Besides, our results are only applicable to solitary HCC less than 5 cm. Finally, we did not perform survival analysis as the prognostic role of tumor differentiation in HCC has been well established [29, 32]. However, further large-scale multi-center studies enrolling patients with more variegated etiologies of underlying liver diseases and adequate patient follow-up are warranted to validate and refine our findings.

In conclusion, in patients with solitary HCC ≤ 5 cm, we constructed and validated an easy-to-use *HCC differentiation score* based on two EOB-MR imaging features (marked HBP hypointensity and the LR-M category) and serum AFP level. This scoring system allowed accurate assessment of poor tumor differentiation in the preoperative setting and thus might be useful for prompting neo-adjuvant therapy and tailored treatment selection among resection, transplant, and ablation.

## Supplementary Information

**Additional file 1. **Supplementary materials.

## Data Availability

The datasets used and analyzed during the current study are available from the corresponding author on reasonable request.

## Abbreviations

ADC: Apparent diffusion coefficient
AFP: Alpha-fetoprotein
ALB: Albumin
ALT: Alanine aminotransferase
AP: Arterial phase
AST: Aspartate aminotransferase
AUC: Area under the receiver operating characteristic curve
CA199: Carbohydrate antigen 199
DWI: Diffusion-weighted imaging
EOB-MRI: Gadoxetate disodium-enhanced magnetic resonance imaging
HBP: Hepatobilliary phase
HCC: Hepatocellular carcinoma
ICC: Intraclass correlation coefficient
IQR: Interquartile range
LI-RADS/LR: Liver imaging reporting and data system
MD: Moderately differentiated
NPV: Negative predictive value
OR: Odds ratio
PD: Poorly differentiated
PPV: Positive predictive value
PVP: Portal venous phase
T2WI: T2-weighted imaging
TBIL: Total bilirubin
TP: Transitional phase
WD: Well differentiated

## References

[1] Sung H, Ferlay J, Siegel RL (2021). Global cancer statistics 2020: GLOBOCAN estimates of incidence and mortality worldwide for 36 cancers in 185 countries. CA Cancer J Clin.

[2] Kulik L, El-Serag HB (2019). Epidemiology and management of hepatocellular carcinoma. Gastroenterology.

[3] Ruff SM, Rothermel LD, Diggs LP (2020). Tumor grade may be used to select patients with multifocal hepatocellular carcinoma for resection. HPB (Oxford).

[4] Decaens T, Roudot-Thoraval F, Badran H (2011). Impact of tumour differentiation to select patients before liver transplantation for hepatocellular carcinoma. Liver Int.

[5] Lu Z, Sun Z, Liu C (2021). Prognostic nomogram for hepatocellular carcinoma with radiofrequency ablation: a retrospective cohort study. BMC Cancer.

[6] Choi JW, Lee JM, Kim SJ (2013). Hepatocellular carcinoma: imaging patterns on gadoxetic acid-enhanced MR Images and their value as an imaging biomarker. Radiology.

[7] Haimerl M, Utpatel K, Götz A (2021). Quantification of contrast agent uptake in the hepatobiliary phase helps to differentiate hepatocellular carcinoma grade. Sci Rep.

[8] Peng Z, Jiang M, Cai H (2016). Gd-EOB-DTPA-enhanced magnetic resonance imaging combined with T1 mapping predicts the degree of differentiation in hepatocellular carcinoma. BMC Cancer.

[9] Huang K, Dong Z, Cai H (2019). Imaging biomarkers for well and moderate hepatocellular carcinoma: preoperative magnetic resonance image and histopathological correlation. BMC Cancer.

[10] Witjes CDM, Willemssen FEJA, Verheij J (2012). Histological differentiation grade and microvascular invasion of hepatocellular carcinoma predicted by dynamic contrast-enhanced MRI. J Magn Reson Imaging.

[11] Rong D, Liu W, Kuang S (2021). Preoperative prediction of pathologic grade of HCC on gadobenate dimeglumine-enhanced dynamic MRI. Eur Radiol.

[12] Dagogo-Jack I, Shaw AT (2018). Tumour heterogeneity and resistance to cancer therapies. Nat Rev Clin Oncol.

[13] Friemel J, Rechsteiner M, Frick L (2015). Intratumor heterogeneity in hepatocellular carcinoma. Clin Cancer Res.

[14] CT/MRI liver imaging reporting and data system version 2018. American College of Radiology Web site. https://www.acr.org/Clinical-Resources/Reporting-andData-Systems/LI-RADS/CTMRI-LI-RADS-v2018. Accessed 1 Dec 2018.

[15] Chou Y, Lao I, Hsieh P (2019). Gadoxetic acid-enhanced magnetic resonance imaging can predict the pathologic stage of solitary hepatocellular carcinoma. World J Gastroenterol.

[16] WHO Classification of tumors: digestive system tumours. 5th edn. Lyon, France. Available https://whobluebooks.iarc.fr/publications/index.php. Accessed 19 Jan 2021

[17] Ringe KI, Husarik DB, Sirlin CB, Merkle EM (2010). Gadoxetate disodium-enhanced MRI of the liver: part 1, protocol optimization and lesion appearance in the noncirrhotic liver. AJR Am J Roentgenol.

[18] Shin J, Lee S, Kim SS (2021). Characteristics and early recurrence of hepatocellular carcinomas categorized asLR-M: comparison with those categorized as LR-4 or 5. J Magn Reson Imaging.

[19] Choi SY, Kim SH, Park CK (2018). Imaging features of gadoxetic acid-enhanced and diffusion-weighted MR imaging for identifying cytokeratin 19-positive hepatocellular carcinoma: a retrospective observational study. Radiology.

[20] Feng Z, Li H, Zhao H (2021). Preoperative CT for characterization of aggressive macrotrabecular-massive subtype and vessels that encapsulate tumor clusters pattern in hepatocellular carcinoma. Radiology.

[21] An C, Park S, Chung YE (2017). Curative resection of single primary hepatic malignancy: liver imaging reporting and data system category LR-M portends a worse prognosis. AJR Am J Roentgenol.

[22] Montal R, Andreu-Oller C, Bassaganyas L (2019). Molecular portrait of high alpha-fetoprotein in hepatocellular carcinoma: implications for biomarker-driven clinical trials. Br J Cancer.

[23] Jiang H, Wei J, Fu F (2022). Predicting microvascular invasion in hepatocellular carcinoma: a dual-institution study on gadoxetate disodium-enhanced MRI. Liver Int.

[24] Mule S, Galletto PA, Tenenhaus A (2020). Multiphase liver MRI for identifying the macrotrabecular-massive subtype of hepatocellular carcinoma. Radiology.

[25] Kang HJ, Kim H, Lee DH (2021). Gadoxetate-enhanced MRI features of proliferative hepatocellular carcinoma are prognostic after surgery. Radiology.

[26] Ji GW, Zhu FP, Xu Q (2020). Radiomic features at contrast-enhanced CT Predict recurrence in early stage hepatocellular carcinoma: a multi-institutional study. Radiology.

[27] Mehta N, Heimbach J, Harnois DM (2017). Validation of a risk estimation of tumor recurrence after transplant (RETREAT) score for hepatocellular carcinoma recurrence after liver transplant. JAMA Oncol.

[28] Zhu AX, Kang YK, Yen CJ (2019). Ramucirumab after sorafenib in patients with advanced hepatocellular carcinoma and increased α-fetoprotein concentrations (REACH-2): a randomised, double-blind, placebo-controlled, phase 3 trial. Lancet Oncol.

[29] Shinkawa H, Tanaka S, Kabata D (2021). The prognostic impact of tumor differentiation on recurrence and survival after resection of hepatocellular carcinoma is dependent on tumor size. Liver Cancer.

[30] Akateh C, Black SM, Conteh L (2019). Neoadjuvant and adjuvant treatment strategies for hepatocellular carcinoma. World J Gastroenterol.

[31] Heimbach JK, Kulik LM, Finn RS (2018). AASLD guidelines for the treatment of hepatocellular carcinoma. Hepatology.

[32] Liao S, Su T, Jeng Y (2019). Clinical manifestations and outcomes of patients with sarcomatoid hepatocellular carcinoma. Hepatology.

