# Supplementary material for: Predicting histologic differentiation of solitary hepatocellular carcinoma up to 5 cm on gadoxetate disodium-enhanced MRI
Source: Insights Imaging. 2023 Jan 8;14:3. doi: 10.1186/s13244-022-01354-w (PMC9826771; doi:10.1186/s13244-022-01354-w)

ELECTRONIC SUPPLEMENTARY MATERIAL

Predicting histologic differentiation of solitary hepatocellular carcinoma up to 5 cm on gadoxetate disodium-enhanced MRI

Table of Contents

Supplementary A1 MRI technique 1

Table S1 MRI sequences and parameters 2

Table S2 Definitions of EOB-MRI features...4

Table S3 Baseline clinical characteristics of patients in the training set and validation set..8

Table S4 Interobserver agreement of imaging features..10

Fig. S1 Calibration plots for predicting HCC differentiation on the training set and validation set...12

Fig. S2 Decision curves for predicting HCC differentiation on the training set and validation set 13

Insights Imaging (2022) Yang T, Wei H, We Y et al.

Supplementary A1

MRI technique

All gadoxetate disodium-enhanced MR imaging (EOB-MRI) were acquired using four 3.0-T systems (MAGNETOM Skyra, Siemens Healthineers; Discovery MR 750, GE Healthcare; SIGNA™ Architect, GE Healthcare; and SIGNA™ Premier, GE Healthcare) and a 1.5-T system (uMR588, United Imaging Healthcare). In-phase and opposed phase T1-weighted imaging and diffusion-weighted imaging (b values: 0, 50, 500, 800, 1000, and 1200 s/mm^2 [Siemens MAGNETOM Skyra]; 0, 200, 800, and 1000 s/mm^2 [GE Discovery MR 750]) with apparent diffusion coefficient maps were obtained. Precontrast images were acquired with a fat-suppressed T1-weighted three-dimensional gradient echo (GRE) sequence. 0.025 mmol/kg of gadoxetate disodium (Xianai®; Zhengdatianqing Pharmaceutical Group or Primovist®; Bayer Schering Pharma AG) was injected intravenously at a flow rate of 1-2 ml/s, followed by 20-30 ml saline. Dynamic imaging was carried out in the late arterial phase, portal venous phase (60 s), and transitional phase (3 minutes) using the same sequences used for precontrast images. Hepatobiliary phase (HBP) images were achieved 20 minutes after start of contrast media injection. Details of the MRI sequences and parameters are shown in **Table S1.**

Table S1 Sequences and parameters of gadoxetate disodium-enhanced MRI

Vendor	TR (ms)	TE (ms)	Flip angle (°)	ST (mm)	Spacing (mm)	Matrix size	FOV (mm^2)	Acquisition Time (s)
T2-weighted 2D FSE†								
1	2160	100	160	6	1.8	320×288	433×433	36
2	6315	78	111	6	2	288×244	360×280	Respiratory gating
3	2400	85	111	7	2	320×192	380×304	34
4	2200	85	111	7	2	320×224	304×380	47
5	2600	99.2	90	6.5	1.5	256×168	427×320	39
Diffusion-weighted imaging†								
1	5600	68	90	6	1.8	100×76	380×289	233
2	9230	Minimum	90	6	2	128 × 128	360× 380	Respiratory gating
3	5000	Minimum	90	7	2	160×128	380×342	Respiratory gating
4	5000	Minimum	90	7	2	120 × 240	380× 380	Respiratory gating
5	3350	77	90	6.5	10	128×92	320×400	Respiratory gating
In- and opposed-phase T1-weighted imaging								
1	81	2.72/1.4	70	6	1.8	352×286	400×325	24
2	150	2.5/1.3	70	6	2	288×192	420×420	31
3	233.8	2.3/1.1	55	7	2	160×288	380×323	18
4	146.8	2.3/1.1	55	7	2	320×192	342×380	16
5	117.6	2.27	60	6.5	1.3	256×174	320×400	29
Dynamic T1-weighted 3D GRE†								
1	3.95	1.92	9	2.5	-	352×256	400×296	14

2	4.1	1.9	15	2	-	512×512	380× 300	15
3	3.9	1.7	15	3	-	320×240	380×380	15
4	3.2	1.4	15	2.4	-	320×240	380× 380	15
5	4.2	1.88	10	2.5	-	256×154	255×400	13

Vendor 1, Siemens MAGNETOM Skyra (18-channel body array coil); Vendor 2, GE Discovery MR 750 (16-channel phased-array torsor coil); Vendor 3, GE SIGNA™ Architect 3.0 Tesla (30-channel body anterior coil); Vendor 4, GE SIGNA™ Premier 3.0 Tesla (30-channel body anterior coil); Vendor 5, uMR588 1.5 Tesla (6-channel body anterior coil).

MRI, magnetic resonance imaging; IP, in-phase; OP, opposed-phase; 3D, three-dimensional; GRE, gradient recall echo; 2D, two-dimensional; FSE, fast spin-echo; RG, respiratory gating.

†Images were acquired under fat suppression.

Table S2 Definitions of EOB-MRI features

MRI feature†	Definition
**Tumor diameter or Size**	Largest outer-edge-to-outer-edge dimension of a liver observation [1]
**Non-rim arterial phase hyperenhancement**	Nonrim-like enhancement of the liver observation in arterial phase unequivocally greater in whole or in part than liver [1]
**Non-peripheral "washout"**	Spatially defined subtype of “washout” in which apparent washout is not most pronounced in the periphery of the observation. The “washout” may have a range of appearances such as diffuse and homogeneous, diffuse and heterogeneous, focal, scattered (patchy, spotty), nodule-in-nodule, or mosaic. [1]
**Enhancing "capsule"**	Smooth, uniform, sharp border around most or all of a liver observation, unequivocally thicker or more conspicuous than fibrotic tissue around background nodules, and visible as enhancing rim in portal venous phase or transitional phase [1]
**Corona enhancement**	Periobservational enhancement in late arterial phase or early portal venous phase attributable to venous drainage from tumor [1]
**Fat sparing in solid mass**	Relative paucity of fat in solid mass relative to steatotic liver OR in inner nodule relative to steatotic outer nodule [1]
**Diffusion restriction** ‡	Signal intensity of the liver observation on diffusion-weighted imaging, not attributable solely to T2 shine-through, unequivocally higher than liver and/or apparent diffusion coefficient unequivocally lower than liver [1]
**Mild-moderate T2 hyperintensity**	Signal intensity of the liver observation on T2-weighted imaging mildly or moderately higher than liver and similar to or less than non-iron-overloaded spleen [1]
**Iron sparing in solid mass**	Paucity of iron in solid mass relative to iron-overloaded liver OR in inner nodule relative to siderotic outer nodule [1]

**Transitional phase hypointensity**	Signal intensity of the liver observation in the transitional phase unequivocally less, in whole or in part, than liver [1]
**Hepatobiliary phase hypointensity**	Signal intensity of the liver observation in the hepatobiliary phase unequivocally less, in whole or in part, than liver [1]
**Non-enhancing "capsule"**	Capsule appearance not visible as an enhancing rim [1]
**Nodule-in-nodule**	Presence of smaller inner nodule within and having different imaging features than larger outer nodule [1]
**Mosaic architecture**	Presence of randomly distributed internal nodules or compartments, usually with different imaging features [1]
**Fat in mass, more than adjacent liver**	Excess fat within a mass, in whole or in part, relative to adjacent liver [1]
**Blood products in mass**	Intralesional or perilesional hemorrhage in the absence of biopsy, trauma or intervention [1]
**Iron in mass, more than liver**	Excess iron in a mass relative to background liver [1]
**Marked T2 hyperintensity**	Signal intensity of the liver observation on T2 weighed-imaging markedly higher than liver and similar to bile ducts and other fluid-filled structures [1]
**Hepatobiliary phase isointensity**	Signal intensity of the liver observation in the hepatobiliary phase nearly identical to liver [1]
**Tumor in vein**	Presence of unequivocal enhancing soft tissue in vein [1]
**Rim arterial phase hyperenhancement**	Presence of arterial phase enhancement most pronounced in observation periphery [1]
**Peripheral "washout"**	Presence of apparent washout most pronounced in observation periphery [1]
**Delayed central enhancement**	Central area of progressive postarterial phase enhancement [1]
**Targetoid TP or HBP appearance**	Concentric pattern in TP or HBP characterized by moderate-to-marked hypointensity in observation periphery with milder hypointensity in center [1]
**Infiltrative appearance**	Liver observation with non-circumscribed margin (indistinct transition) [1]
**Marked diffusion restriction**	Increased signal intensity of the liver observation at diffusion-weighted imaging in relative to the spleen, not solely attributable to

	T2-weighted imaging shine-through effect [1]
**Necrosis or severe ischemia**	Presence of unequivocal intralesional necrosis or severe ischemia [1]
**Liver surface retraction**	Presence of focal flattening or concavity of the normally convex hepatic contour [2]
**Adjacent biliary dilatation**	Presence of dilated bile ducts adjacent to the tumor border in any imaging plane
**Bilobar involvement**	Bilobar involvement of definite HCC on gadoxetate disodium-enhanced MR imaging
**Internal artery**	Presence of discrete arterial enhancement within the tumor [3]
**Non-smooth tumor margin**	Presence of non-nodular tumors with irregular contour that had budding portion at the periphery [4]
**Peritumoral hypointensity on PVP**	Presence of wedge-shaped or flame-like hypointense area adjacent to the tumor border on portal venous phase images
**Peritumoral hypointensity on TP**	Presence of wedge-shaped or flame-like hypointense area adjacent to the tumor border on transitional phase images
**Peritumoral hypointensity on HBP**	Presence of wedge-shaped or flame-like hypointense area adjacent to the tumor border on hepatobiliary phase images [4]
**Marked HBP hypointensity**	Signal intensity of the tumor in the hepatobiliary phase is lower than that of liver and similar to or lower than that of intrahepatic vessels
**Complete capsule**	Presence of non-disrupted "capsule" in all imaging planes [5]
**Peritumoral hyperintensity on T2WI**	Presence of a round expanding nodule with a distinct margin [6]
**Non-hypervascular HBP hypointense nodules**	Presence of solid nodules with low signal intensity on HBP greater than 3.5 mm in diameter, which do not show higher signal intensity than that of the spleen on heavily T2-weighted images [7]

EOB-MRI, gadoxetate disodium-enhanced magnetic resonance imaging; HCC, hepatocellular carcinoma; PVP, portal venous phase; HBP, hepatobiliary phase; AP, arterial phase; TP, transitional phase; IP, in-phase; OP, opposed-phase; DWI,

diffusion-weighted imaging; T2WI, T2-weighted imaging; T1WI, T1-weighted imaging; LI-RADS/LR, Liver Imaging Reporting and Data System.

†LI-RADS v2018 features correlated with growth or ultrasound visibility were not assessed due to lack of prior or concurrent ultrasound examinations.

References

1. CT/MRI Liver Imaging Reporting and Data System version 2018 (2018) Available via https://www.acr.org/Clinical-Resources/Reporting-and-Data-Systems/LI-RADS/CT-MRI-LI-RADS-v2018. Accessed 25 Oct 2021
2. Blachar A, Federle MP, Sosna J (2009) Liver lesions with hepatic capsular retraction. Semin Ultrasound CT MR 30:426-435
3. Renzulli M, Brocchi S, Cucchetti A et al (2016) Can current preoperative imaging be used to detect microvascular invasion of hepatocellular carcinoma? Radiology 279:432-442
4. Lee S, Kim SH, Lee JE, Sinn DH, Park CK (2017) Preoperative gadoxetic acid-enhanced MRI for predicting microvascular invasion in patients with single hepatocellular carcinoma. J Hepatol 67:526-534
5. Lei Z, Li J, Wu D et al (2016) Nomogram for preoperative estimation of microvascular invasion risk in hepatitis B virus-related hepatocellular carcinoma within the Milan criteria. JAMA Surg 151:356-363
6. Rhee H, Chung T, Yoo JE et al (2020) Gross type of hepatocellular carcinoma reflects the tumor hypoxia, fibrosis, and stemness-related marker expression. Hepatol Int 14(2):239-248.

7. Toyoda H, Kumada T, Tada T, et al (2013) Non-hypervascular hypointense nodules detected by Gd-EOB-DTPA-enhanced MRI are a risk factor for recurrence of HCC after hepatectomy. J Hepatol 58(6):1174-1180.

Table S3 Baseline clinical characteristics of patients in the training set and validation set

	All (n=182)	Training set (n=128)	Validation set (n=54)	*P* value
Patient demographics				
Age, years	52.9±11.2	52.8±11.5	53.2±10.6	0.517
Sex				0.680
Male	145 (79.7)	103 (80.5)	42 (77.8)	
Female	37 (20.3)	25 (19.5)	12 (22.2)	
Underlying liver diseases				
Chronic hepatitis B	171 (94.0)	119 (93.0)	52 (96.3)	0.603
Chronic hepatitis C	1 (0.5)	0 (0.0)	1 (1.9)	...
Chronic hepatitis B and C	2 (1.1)	2 (1.6)	0 (0.0)	...
Alcohol	2 (1.1)	2 (1.6)	0 (0.0)	...
NAFLD	2 (1.1)	1 (0.8)	1 (1.9)	...
Others	4 (2.2)	4 (3.1)	0 (0.0)	...
Cirrhosis	109 (59.9)	79 61.7)	30 (55.6)	0.438
Child-Pugh stage				0.556
A	179 (98.4)	125 (97.7)	54 (100.0)	
B	3 (1.6)	3 (2.3)	0 (0.0)	
Tumor marker				
AFP, ng/mL				0.134
≤ 400	146 (80.2)	99 (77.3)	47 (87.0)	
> 400	36 (19.8)	29 (22.7)	7 (13.0)	
CA199, U/ml				0.309
≤ 30	143 (78.6)	98 (76.6)	45 (83.3)	
> 30	39 (21.4)	30 (23.4)	9 (16.7)	
CEA, ng/ml				0.035*
≤ 5	172 (94.5)	118 (92.2)	54 (100.0)	
> 5	10 (5.5)	10 (7.8)	0 (0.0)	
Laboratory index				
TBIL, umol/L				0.493
≤ 40	180 (98.9)	126 (98.4)	54 (100.0)	

> 40	2 (1.1)	2 (1.6)	0 (0.0)	
ALT, U/L				0.465
≤ 35	107 (58.8)	90 (70.3)	35 (64.8)	
> 35	75 (41.2)	38 (29.7)	19 (35.2)	
AST, U/L				0.641
≤ 35	127 (69.8)	88 (68.8)	39 (72.2)	
> 35	55 (30.2)	40 (31.3)	15 (27.8)	
ALB, g/L				0.657
≥ 40	142 (78.0)	101 (78.9)	41 (75.9)	
< 40	40 (22.0)	27 (21.1)	13 (24.1)	
PLT, 10^9/L				0.341
≥ 125	84 (46.2)	62 (48.4)	22 (40.7)	
< 125	98 (53.8)	66 (51.6)	32 (59.3)	
PT, s				0.784
≤ 12.8	157 (86.3)	111 (86.7)	46 (85.2)	
> 12.8	25 (13.7)	17 (13.3)	8 (14.8)	

Data are expressed as n (%) or median (interquartile range)

HBV; hepatitis B virus; AFP, a-fetoprotein; CA199, Carbohydrate antigen 199; CEA, carcinoembryonic antigen; TBIL, total bilirubin; ALT,alanine aminotransferase; AST, aspartate aminotransferase; ALB, albumin; PLT, platelet count; PT, prothrombin time. Continuous variables are presented as mean ± standard deviation. These variables were compared using t-test or nonparametric Mann-Whitney tests. Categorical variables are the number of patients, with percentages in parentheses. These variables were compared using the chi-square or Fisher exact test.

*Variables are statistically significant.

Table S4 Interobserver agreement of imaging features

	Reviewer 1	Reviewer 2	*Kappa* value
LI-RADS v2018 feature†			
Size, cm	2.64±1.03	2.70±1.06	0.950 (0.933, 0.962)*
Non-rim arterial phase hyperenhancement	153 (84.1)	166 (91.2)	0.373 (0.182, 0.565)
Non-peripheral "washout"	22 (12.1)	18 (9.9)	0.733 (0.632, 0.833)
Enhancing "capsule"	132 (72.5)	113 (62.1)	0.272 (0.130, 0.415)
Corona enhancement	63 (34.6)	57 (31.3)	0.354 (0.211, 0.497)
Fat sparing in solid mass	19 (10.4)	10 (5.5)	0.443 (0.211, 0.674)
Diffusion restriction	181 (99.5)	182 (100.0)	0.421 (0.274, 0.568)
Mild-moderate T2 hyperintensity	175 (96.2)	179 (98.4)	0.181 (-0.153, 0.515)
Iron sparing in solid mass	37 (20.3)	40 (22.0)	0.424 (0.264, 0.583)
Transitional phase hypointensity	161 (88.5)	173 (95.1)	0.355 (0.130, 0.581)
Hepatobiliary phase hypointensity	170 (93.4)	175 (96.2)	0.406 (0.072, 0.739)
Non-enhancing "capsule"	35 (19.2)	23 (12.6)	0.239 (0.056, 0.422)
Nodule-in-nodule	42 (23.1)	60 (33.0)	0.327 (0.181, 0.473)
Mosaic architecture	55 (30.2)	32 (17.6)	0.039 (-0.101, 0.180)
Fat in mass, more than adjacent liver	84 (46.2)	76 (41.8)	0.288 (0.148, 0.427)
Blood products in mass	30 (16.5)	35 (19.2)	0.532 (0.372, 0.693)
Iron in mass, more than liver	4 (2.2)	3 (1.6)	0.272 (-0.171, 0.715)
Marked T2 hyperintensity	4 (2.2)	2 (1.1)	0.323 (-0.166, 0.813)
Hepatobiliary phase isointensity	7 (3.8)	6 (3.3)	0.311 (-0.039, 0.662)
Tumor in vein	4 (2.2)	4 (2.2)	1.000 (1.000, 1.000)
Rim arterial phase hyperenhancement	19 (10.4)	5 (2.7)	0.390 (0.147, 0.633)
Peripheral "washout"	12 (6.6)	1 (0.5)	0.126 (-0.116, 0.369)
Delayed central enhancement	4 (2.2)	4 (2.2)	-0.022 (-0.038, -0.007)
Targetoid TP or HBP appearance	13 (7.1)	0 (0.0)	0 (0.000, 0.000)
Infiltrative appearance	22 (12.1)	18 (9.9)	0.327 (0.120, 0.534)
Marked diffusion restriction	67 (36.8)	66 (36.3)	0.301 (0.158, 0.444)
Necrosis or severe ischemia	28 (15.4)	32 (17.6)	0.402 (0.226, 0.577)
Liver surface retraction	6 (3.3)	3 (1.6)	0.205 (-0.160, 0.570)
Adjacent biliary dilatation	2 (1.1)	3 (1.6)	-0.013 (-0.026, -

			0.001)
LR-M category	7 (3.8)	7 (3.8)	0.406 (0.072, 0.739)
Other imaging feature			
Radiologic cirrhosis	112 (61.5)	111 (61.0)	0.572 (0.449, 0.694)
Bilobar involvement	13 (7.1)	15 (8.2)	0.459 (0.219, 0.698)
Internal artery	36 (19.8)	25 (13.7)	0.198 (0.028, 0.367)
Non-smooth tumor margin	82 (45.1)	107 (58.8)	0.406 (0.279, 0.533)
Peritumoral hypointensity in PVP	25 (13.7)	40 (22.0)	0.352 (0.185, 0.519)
Peritumoral hypointensity in TP	26 (14.3)	35 (19.2)	0.275 (0.100, 0.449)
Peritumoral hypointensity in HBP	49 (26.9)	51 (28.0)	0.449 (0.304, 0.593)
Marked HBP hypointensity	53 (29.1)	52 (28.6)	0.451 (0.309, 0.594)
Complete capsule	92 (50.5)	82 (45.1)	0.385 (0.252, 0.518)
Single nodular type growth	55 (30.2)	59 (32.4)	0.770 (0.694, 0.847)
Peritumoral hyperintensity on T2WI	16 (8.8)	22 (12.1)	0.355 (0.145, 0.566)
Non-hypervascular HBP hypointense nodules	54 (29.4)	49 (26.9)	0.445 (0.302, 0.589)

Data are expressed as the frequencies of MRI features, with percentages in parentheses.

Kappa value: 0.000-0.200 indicates slight consistency, 0.201-0.400 indicates fair consistency, 0.401-0.600 indicates moderate consistency, 0.601-0.800 indicates substantial consistency and 0.801-1.000 indicates almost perfect consistent.

EOB-MRI, gadoxetate disodium-enhanced magnetic resonance imaging; LI-RADS/LR, Liver Imaging Reporting and Data System; HCC, hepatocellular carcinoma; WD, well differentiated; MD, moderately differentiated; PD, poorly differentiated; HBV; T2WI, T2-weighted imaging; PVP, portal venous phase; TP, transitional phase; HBP, hepatobiliary phase.

[*]Variable was calculated using intraclass correlation coefficient.

[†]LI-RADS v2018 features correlated with growth or ultrasound visibility were not assessed due to lack of prior and concurrent ultrasound examinations.

Fig. S1 Calibration plots for predicting HCC differentiation on the training set (a) and validation set (b)**.**

(a)

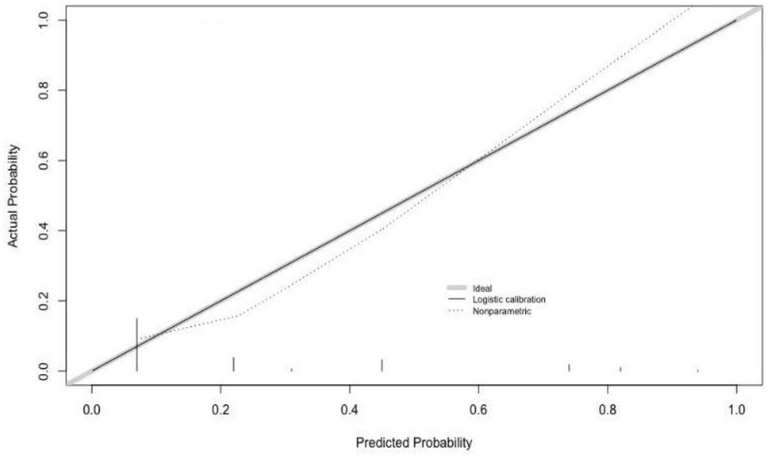

(b)

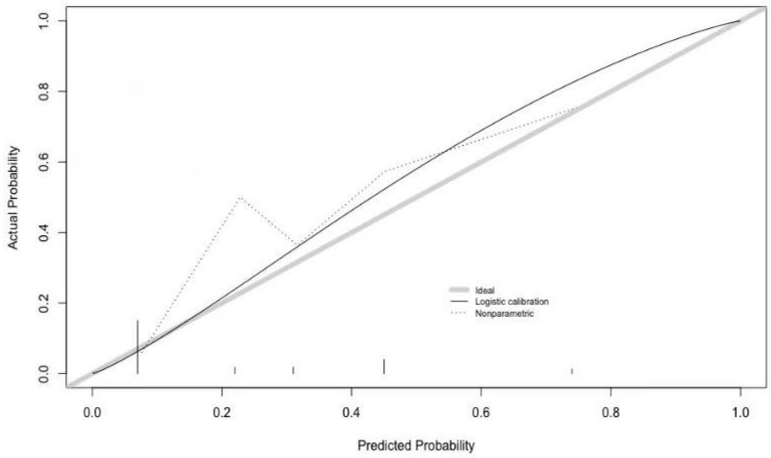

Fig. S2 Decision curves for predicting HCC differentiation on the training set (a) and validation set (b).

(a)

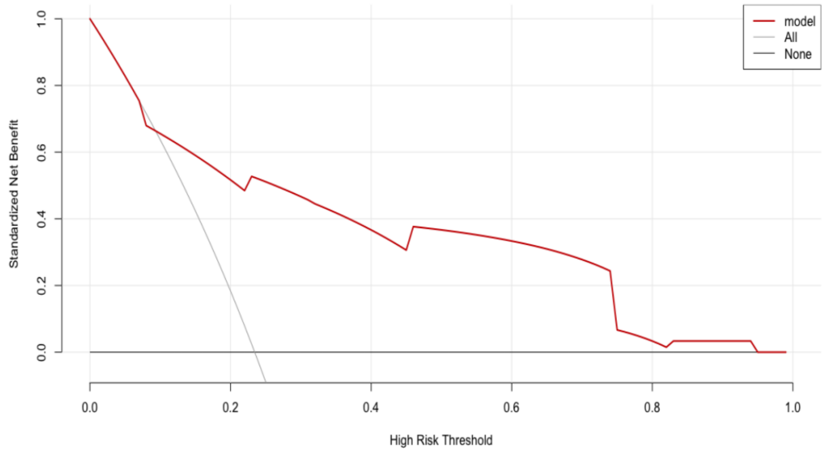

(b)

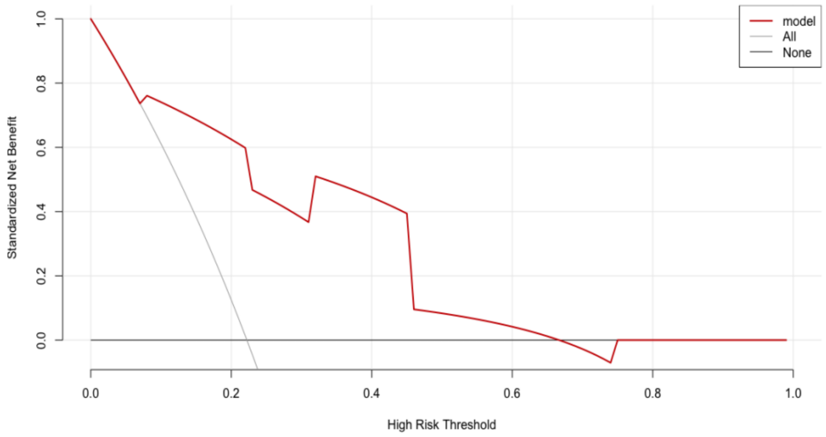

Supplement: Supplementary file 1 — Additional file 1. Supplementary materials. [file 13244_2022_1354_MOESM1_ESM.pdf]
